# Effect of a high-fat diet and chromium on hormones level and Cr retention in rats

**DOI:** 10.1007/s40618-021-01677-3

**Published:** 2021-09-22

**Authors:** A. Stępniowska, K. Tutaj, J. Juśkiewicz, K. Ognik

**Affiliations:** 1 Department of Biochemistry and Toxicology, Faculty of Animal Sciences and Bioeconomy, University of Life Sciences in Lublin, Akademicka 13, 20-950 Lublin, Poland; 2 Division of Food Science, Institute of Animal Reproduction and Food Research of the Polish Academy of Sciences, Tuwima 10, 10-748 Olsztyn, Poland

**Keywords:** Chromium, Rat, Hormone, High-fat diet, Cr digestibility

## Abstract

**Aims:**

The aim of the study was to determine how the administration of a high-fat diet supplemented with various forms of chromium to rats affects accumulation of this element in the tissues and levels of leptin, ghrelin, insulin, glucagon, serotonin, noradrenaline and histamine, as well as selected mineral elements.

**Methods:**

The experiment was conducted on 56 male Wistar rats, which were divided into 8 experimental groups. The rats received standard diet or high fat diet (HFD) with addition of 0.3 mg/kg body weight of chromium(III) picolinate (Cr-Pic), chromium(III)-methioninate (Cr-Met), or chromium nanoparticles (Cr-NP).

**Results:**

Chromium in organic forms was found to be better retained in the body of rats than Cr in nanoparticles form. However, Cr-Pic was the only form that increased the insulin level, which indicates its beneficial effect on carbohydrate metabolism. In blood plasma of rats fed a high-fat diet noted an increased level of serotonin and a reduced level of noradrenaline. The addition of Cr to the diet, irrespective of its form, also increased the serotonin level, which should be considered a beneficial effect. Rats fed a high-fat diet had an unfavourable reduction in the plasma concentrations of Ca, P, Mg and Zn. The reduction of P in the plasma induced by supplementation with Cr in the form of Cr-Pic or Cr-NP may exacerbate the adverse effect of a high-fat diet on the level of this element.

**Conclusion:**

A high-fat diet was shown to negatively affect the level of hormones regulating carbohydrate metabolism (increasing leptin levels and decreasing levels of ghrelin and insulin).

## Introduction

Diet, particularly a diet rich in fats, plays a key role in the development of civilization diseases such as diabetes, cardiovascular disease, and cancer [1]. Studies on animal models fed a high-fat diet (HFD) have shown many adverse metabolic changes, such as hypertriglyceridemia, hyperinsulinemia and glucose intolerance [2]. In addition, a long-term HFD is associated with the risk of obesity. Obese animals have been shown to have increased levels of leptin, the hormone encoded by the obesity (*ob*) gene. In physiological conditions, the plasma leptin concentration is proportional to the amount of adipose tissue in the body, and thus concentrations of this hormone are higher in obese individuals than in those with normal body weight [3]. Leptin has many functions in the organism, it decreases appetite and increases energy consumption, thereby acting as a long-term regulator of body weight [3, 4]. The hyperleptinemia observed in obese humans and animals suggests that their organisms are not sensitive to endogenous leptin [5]. Ghrelin has the opposite effect on the organism to leptin. This hormone is secreted by the stomach and acts as a meal initiator. Ghrelin peripherally and centrally administered to rats increased food intake, induced weight gain, stimulated secretion of gastric juice, and improved gastric motility [6]. Olszanecka-Glinianowicz et al. [7], in a study in obese women, noted a reduction in fasting ghrelin secretion, as well as an increase in its levels as a result of weight loss. A high-fat diet also causes impairment of insulin secretion, i.e. insulin resistance. Insulin increases expression of the *ob* gene, and thus the release of leptin from the adipocytes [8]. According to Posey et al. [9], diet-induced obesity involves the acquisition of functional resistance of the central nervous system (CNS) to insulin and leptin, which in turn leads to pathological body weight gain. Because insulin and leptin can use the same intracellular signalling pathways, neuronal resistance to these hormones may involve the same or similar mechanisms [10].

Energy homeostasis is also regulated by neurotransmitters such as serotonin (5-HT) and dopamine [11, 12]. Changes in eating behaviour in obese individuals are due to changes in serotonin and dopamine secretion [13, 14]. Increased serotonin levels cause a feeling of satiety and are conducive to a reduction in food intake. Research by Haleem and Khalid [15] showed that a high-fat diet reduces the level of 5-HT in the hypothalamus and increases levels of 5-HT and 5-HIAA (5-hydroxyindoleacetic acid) in the hippocampus of rats. Both inhibition and stimulation of serotonin and dopamine secretion are associated with differences in feeding behaviour, stimuli to eat, and energy expenditure [16].

A high-fat diet also indirectly affects mineral metabolism by disturbing normal hormone secretion. Iron is important for glucose-stimulated insulin secretion, but excessive iron induces oxidative stress and increases apoptosis in pancreatic β cells. The mechanisms whereby excess iron contributes to type 2 diabetes (T2D) are not fully understood but most likely involve insulin resistance and impairment of the function of pancreatic β cells [17]. On the other hand zinc increases uptake of 5-HT in the corpus callosum, cingulate cortex, and raphe nucleus of the brain of rats [18].

The adverse effect of a high-fat diet on the organism can be neutralised by adding chromium to the diet [19]. This element is involved in glucose metabolism, in part as a component of glucose tolerance factor (GTF). Chromium activates insulin receptor and increases insulin-dependent glucose uptake into cells. Furthermore, Cr reduces the leptin level in the blood [20]. Thus, chromium is a popular dietary supplement in the treatment of type 2 diabetes and the promotion of weight loss [21]. The addition of Cr to the diet also increases secretion of neurotransmitters such as serotonin and noradrenalin [22].

Both absorption of Cr and its effects on the organism depend on its chemical form. Inorganic compounds of Cr(III) are poorly absorbed in the gastrointestinal tract, while the organic forms of Cr like chromium picolinate (Cr-Pic), chromium malate or complexes with various amino acids are much more easily digested [23, 24]. Not all organic forms of Cr are absorbed to the same degree. According to Anderson et al. [25], Cr in the form of amino acid complexes is better absorbed than Cr in the form of Cr-Pic. This means that absorption of organic forms of Cr depends on the organic ligand. Cr nanoparticles are also becoming increasingly popular; this includes both metallic Cr nanoparticles and nanoparticles in the form of chemical compounds, such as nano-Cr-Pic. Owing to the small size of nanoparticles, Cr in nano-form may be more digestible than macro-forms. However, knowledge of both the absorption of Cr nanoparticles and their effects on the body is still inadequate. As different forms of Cr are absorbed in varying degrees, their effect on the body may vary as well. We hypothesised that the addition of chromium to a high-fat diet would reduce the negative effect of that diet on the secretion of hormones regulating carbohydrate metabolism and physiologically important neurotransmitters. It was additionally postulated that chromium in nanoparticle form will be more easily digestible and better retained in the body of rats than chromium in organic form, and thus the regulatory effect of this form of Cr on hormonal metabolism will be more efficient. The aim of the study was to determine the how the administration of a high-fat diet supplemented with various forms of chromium to rats affects accumulation of this element in the tissues and levels of leptin, ghrelin, insulin, glucagon, serotonin, noradrenaline and histamine, as well as selected mineral elements.

## Materials and methods

The present study is part of a larger experiment. Experimental design and other physiological results have been published in Ognik et al. [26], Dworzański et al. [27] and Dworzański et al. [28].

### Animals and diets

The experiment was conducted on 56 male Wistar outbred rats (*Rattus norvegicus*, Cmdb:WI). The animals were used in compliance with the European guidelines for the care and use of animal models [29]. The Animal Care and Use Committee in Olsztyn (Poland) approved the experimental protocol (approval no. 04/2019). All efforts were made to minimise the suffering of the experimental animals. At the start of the experiment the rats weighed 131 ± 4.33 g and were randomly assigned to one of eight groups of seven rats each. The animals were kept individually in metabolic cages (Tecniplast Spa, Buguggiate, Italy) under a stable temperature (21–22 °C), a 12-h light:12-h dark cycle, and a ventilation rate of 20 air changes per hour. For 8 weeks the rats had free access to tap water and semi-purified diets, which were prepared and then stored at 4 °C in hermetic containers until the end of the experiment (Table 1). The diets were modifications of a casein diet for laboratory rodents recommended by the American Institute of Nutrition [30]. Two types of diet were used in the experiment: a standard diet (diet S) containing 8% rapeseed oil and 5% cellulose as sources of fat and dietary fibre, respectively, and a high-fat, low-fibre diet (HFD), which was a modification of diet S with 17% lard added in place of maize starch and with cellulose content reduced to 3%. All diets had an identical amount of dietary protein originating from a casein preparation (Lacpol Co., Murowana Goslina, Poland) and DL-methionine, comprising 20% and 0.3% of the diets, respectively. Different chromium sources, i.e. chromium(III) picolinate (Cr-Pic), chromium(III)-methioninate (Cr-Met), and chromium nanoparticles (Cr-NP), were added to the standard and high-fat diets. Chromium picolinate (Cr-Pic; purity > 980 g/kg) was purchased from Sigma-Aldrich Sp. z o.o. (Poznan, Poland). Chromium-methionine complex (Cr-Met) was purchased from Innobio Co., Ltd. (Siheung, Korea). Chromium nanoparticles (Cr-NP) with 99.9% purity, size 60–80 nm, spherical shape, specific surface area 6–8 m^2^/g, bulk density 0.15 g/cm^3^, and true density 8.9 g/cm^3^, was produced and purchased from SkySpring Nanomaterials (Houston, TX, USA).

**Table 1:** Composition of diets fed to rats, %

Ingredient	Diet	
	Standard	High-fat
Casein^a^	20.0	20.0
DL-methionine	0.3	0.3
Cellulose^b^	5.0	3.00
Sucrose	10.0	10.0
Rapeseed oil	8.0	8.0
Lard	–	17.0
Vitamin mixture^c^	1.0	1.0
Mineral mixture^d^	3.5	3.5
Choline chloride	0.2	0.2
Cholesterol	0.3	0.3
Corn starch^f^	51.7	36.7

^a^Casein preparation (LACPOL Co., Murowana Goslina, Poland), crude protein 89.7%, crude fat 0.3%, ash 2.0%, water 8.0%

^b^α-cellulose (SIGMA, Poznan, Poland), main source of dietary fibre

^c^AIN-93G-VM (Reeves, 1997), g kg^−1^ mix: 3.0 nicotinic acid, 1.6 Ca pantothenate, 0.7 pyridoxine–HCl, 0.6 thiamin-HCl, 0.6 riboflavin, 0.2 folic acid, 0.02 biotin, 2.5 vitamin B_12_ (cyanocobalamin, 0.1% in mannitol), 15.0 vitamin E (all-rac-α-tocopheryl acetate, 500 IU g^−1^), 0.8 vitamin A (all-*trans*-retinyl palmitate, 500,000 IU g-1), 0.25 vitamin D_3_ (cholecalciferol, 400,000 IU g-1), 0.075 vitamin K_1_ (phylloquinone), 974.655 powdered sucrose

^d^Mineral mix, g kg^−1^ mix: 357 calcium carbonate anhydrous CaCO_3_, 196 dipotassium phosphate K_2_HPO_4_, 70.78 potassium citrate C_6_H_5_K_3_O_7_, 74 sodium chloride NaCl, 46.6 potassium sulphate K_2_SO_4_, 24 magnesium oxide MgO, 18 microelement mixture^e^, starch to 1 kg = 213.62

^e^Microelement mixture, g kg^−1^ mix: 31 iron (III) citrate (16.7% Fe), 4.5 zinc carbonate ZnCO_3_ (56% Zn), 23.4 manganese (II) carbonate MnCO_3_ (44.4% Mn), copper carbonate CuCO_3_ (55.5% Cu), 0.04 potassium iodide KI, citric acid C_6_H_8_O_7_ to 100 g

^f^Corn starch preparation: crude protein 0.6%, crude fat 0.9%, ash 0.2%, total dietary fibre 0%, water 8.8%

The experimental sources of dietary Cr, chromium(III) picolinate (Cr-Pic), chromium(III)-methioninate (Cr-Met), and chromium nanoparticles (Cr-NP), were added to the diet as an emulsion together with dietary rapeseed oil rather than in the mineral mixture (MX)

The dosage of Cr administered to each rat was 0.3 mg/kg BW and was chosen in accordance with recommendations by the EFSA [31]. To ensure the safety of the individual preparing the experimental diets, the Cr-NP preparation, as well as the other Cr sources in order to maintain comparable conditions, was added to the diet not in a mineral mixture, but as an emulsion together with dietary rapeseed oil.

### Sample collection

The feeding period was 8 weeks. Individual feed consumption of rats were determined. All physiological measurements were made for each animal separately (*n* = 7 for each group). Cr digestibility and utilisation tests (balance tests) were carried out during the study. After a 10 days preliminary period (days 8–17 of experimental feeding), faeces and urine were collected for 5 days (days 18–22) from all rats kept in the metabolic cages. The content of Cr in the diets, drinking water, faeces and urine collected in the balance period was assayed using the methods described below.

At the end of the experiment, the rats were fasted for 12 h and anaesthetized *i.p.* with ketamine and xylazine (K, 100/kg BW; X, 10 mg/kg BW) according to recommendations for anaesthesia and euthanasia of experimental animals. Following laparotomy, blood samples were taken from the caudal vena cava into heparinized tubes, and finally the rats were euthanized by cervical dislocation. The blood plasma was prepared by solidification and low-speed centrifugation (350×*g*, 10 min, 4 °C). Plasma samples were kept frozen at − 70 °C until assayed.

### Laboratory analysis

Chromium was determined colorimetrically by reaction with 1,5-diphenylcarbazide (DPC) in acid solution. About 2 g of the sample was mineralized in a muffle furnace at 550 °C and dissolved in 3 mL of sulphuric acid (5 mol/L). To convert trivalent chromium to its hexavalent state the dissolved ash was transferred from the crucible to a conical flask and heated to the boiling point with potassium permanganate. Potassium permanganate (0.1% in water) was added in 50 µL portions until the oxidation process was completed (the slightly violet colour of the potassium permanganate disappeared until trivalent chromium was present in the solution). The mixture was allowed to cool to room temperature and then transferred to a 50 mL volumetric flask, to which 2 mL of sulphuric acid (5 mol/L) and 0.3 mL of phosphoric acid (98%) were added. Then 24 mL of acidified solution was transferred to a 25 mL volumetric flask containing 1 mL of DPC (0.25% in acetone). The contents were mixed and diluted to mark with distilled water. The absorbance was measured after 15–20 min at 546 nm using a GENESYS 20 spectrophotometer (Thermo Fisher Scientific).

Kits produced by Cell Biolabs, Inc. (San Diego, USA) were used to determine the level of the hormones leptin, ghrelin, insulin, glucagon, serotonin, noradrenaline, and histamine. Content of minerals (Ca, Mg, Fe, and Zn) in the blood samples was determined by flame atomic absorption spectrometry (FAAS).

### Statistical analysis

The results are expressed as means and pooled SEM (standard error of the mean). Two-way analysis of variance (ANOVA) was used to determine the effect of the Cr source (Cr: none, Cr-Pic, Cr-Met, or Cr-NP) and the diet type (D: standard or high-fat diet) and the interaction between these two factors (Cr × D). If the analysis revealed a significant interaction (*P* ≤ 0.05), the differences between treatment groups were then determined by Duncan’s post hoc test at *P* ≤ 0.05. The data were checked for normality prior to the statistical analyses. The statistical analysis was performed using STATISTICA software, version 10.0 (StatSoft Corp., Krakow, Poland).

## Results

### Effect of high-fat diet

Administration of a high-fat diet to rats decreased feed intake (*P* < 0.001), Cr intake from the diet and total Cr intake (*P* < 0.001, both), Cr digestibility and Cr retention (*P* < 0.001, both) and increased Cr excretion in the faeces (*P* = 0.005) compared to the group receiving a standard diet (Table 2). Administration of a high-fat diet to rats decreased the levels of insulin (*P* = 0.017), ghrelin (*P* = 0.005), noradrenaline (*P* = 0.032) and histamine (*P* < 0.001) and increased those of leptin (*P* = 0.019) and serotonin (*P* = 0.048) compared to the group receiving a standard diet (Table 3). The plasma of rats receiving a high-fat diet had lower levels of Ca (*P* = 0.028), P (P < 0.001), Mg (*P* = 0.010) and Zn (*P* = 0.004) than the plasma of rats fed a standard diet (Table 4).

**Table 2:** Chromium excretion patterns in the digestibility and retention test

Item	Diet (D)		Cr form (Cr)				SEM	*P* value		
	Standard	High-fat	None	Cr-Pic	Cr-Met	Cr-NP		D effect	Cr effect	Interaction (Cr × D)
Cr content of diet (mg/kg)	3.31	3.34	1.24^b^	3.99^a^	4.02^a^	4.03^a^	0.161	0.067	< 0.001	0.057
Cr intake from diet (mg/5 day)^A^	0.282^a^	0.253^b^	0.100^b^	0.328^a^	0.322^a^	0.319^a^	0.013	< 0.001	< 0.001	0.082
*otal Cr intake (mg/5d)^B^	0.282^b^	0.253^a^	0.100^b^	0.328^a^	0.322^a^	0.319^a^	0.013	< 0.001	< 0.001	0.082
Excretion of Cr in urine (mg)	0.009	0.010	0.006^b^	0.010^a^	0.011^a^	0.013^a^	0.001	0.373	0.033	0.120
Excretion of Cr in faeces (mg)	0.128^b^	0.174^a^	0.062^c^	0.166^b^	0.164^b^	0.213^a^	0.012	0.005	< 0.001	0.071
Total Cr excretion (mg/5 day)	0.137^b^	0.185^a^	0.068^c^	0.176^b^	0.175^b^	0.226^a^	0.012	0.004	< 0.001	0.011
Cr digestibility (%)	52.23^a^	33.12^b^	39.32^c^	51.60^a^	47.97^b^	31.79^d^	2.329	< 0.001	< 0.001	0.052
Cr retention (%)	48.94^a^	28.43^b^	34.07^b^	48.47^a^	44.18^a^	28.04^c^	2.425	< 0.001	< 0.001	0.066

^A^Cr intake from diet calculated from feed intake data presented in Ognik et al. [26]

Feed intake: diet: standard—17.45 g/day, high-fat—16.17 g/day; Cr form: none—19.9 g/day, Cr-Pic—16.8 g/day, Cr-Met—16.8 g/day, Cr-NP—16.7 g/day [26]

^B^Total Cr intake from diet and water (Cr concentration in water administered to rats 2.9 µg/L)

^a,b,c,d^Mean values within a row with unlike superscript letters were shown to be significantly different (*P*<0.05)

**Table 3:** Content of hormones in the blood plasma of rats

Hormone	Diet (D)		Cr form (Cr)				SEM	*P* value		
	Standard	High-fat	None	Cr-Pic	Cr-Met	Cr-NP		D effect	Cr effect	Interaction (Cr × D)
Leptin, ng/mL	1.163^b^	1.325^a^	1.035^c^	1.341^b^	1.611^a^	0.988^c^	0.057	0.019	< 0.001	0.074
Ghrelin, pg/mL	72.97^a^	62.54^b^	72.26^ab^	62.58^b^	75.67^a^	60.53^b^	3.743	0.005	0.010	0.126
Insulin, mIU/L	60.22^a^	55.48^b^	54.68^b^	61.49^a^	57.43^ab^	57.79^ab^	1.740	0.017	0.034	0.133
Glucagon, pg/mL	4679.7	5180.0	5201.6	4846.6	4587.5	5083.8	137.9	0.073	0.401	0.564
Serotonin, ng/ml	1987.6^b^	2180.7^a^	1859.9^c^	2161.7^b^	2065.7^b^	2249.3^a^	56.53	0.048	0.037	0.225
Noradrenaline, pg/mL	441.76^a^	427.96^b^	445.14^a^	396.19^b^	454.51^a^	443.58^a^	7.125	0.032	0.009	0.147
Histamine, ng/ml	484.46^a^	244.03^b^	568.43^a^	239.73^c^	240.76^c^	408.06^b^	33.76	< 0.001	< 0.001	0.305

^a,b,c^Mean values within a row with unlike superscript letters were shown to be significantly different (*P*<0.05)

**Table 4:** Content of minerals in the blood plasma of rats

Item	Diet (D)		Cr form (Cr)				SEM	*P* value		
	Standard	High-fat	None	Cr-Pic	Cr-Met	Cr-NP		D effect	Cr effect	Interaction (Cr × D)
Ca, mmol/L	2.287^a^	2.172^b^	2.297^ab^	2.113^b^	2.357^a^	2.150^b^	0.031	0.028	0.003	0.312
P, mmol/L	4.329^a^	3.218^b^	4.333^a^	2.981^c^	4.278^a^	3.503^b^	0.129	< 0.001	< 0.001	0.251
Mg, mmol/L	0.831^a^	0.782^b^	0.759^b^	0.841^a^	0.808^ab^	0.818^ab^	0.010	0.010	0.019	0.542
Fe, µmol/L	12.668	13.031	12.474^ab^	11.517^b^	13.199^ab^	14.208^a^	0.453	0.686	0.044	0.298
Zn, µmol/L	19.251^a^	17.860^b^	19.064^ab^	17.708^b^	17.313^b^	20.136^a^	0.319	0.004	< 0.001	0.126

^a,b,c^Mean values within a row with unlike superscript letters were shown to be significantly different (*P*<0.05)

### Effect of form of Cr

Cr content in the diet (*P* < 0.001), Cr intake from the diet (*P* < 0.001), total Cr intake (*P* < 0.001), and excretion of Cr in the urine and faeces (*P* = 0.033, *P* < 0.001, respectively) were all higher in the groups receiving a diet with added Cr, irrespective of its form. Cr % digestibility and Cr % retention were higher in the rats receiving a diet with added Cr-Pic or Cr-Met and lower in rats receiving Cr-NP (*P* < 0.001, all) than in rats receiving a diet without added Cr (Table 2).

The leptin level in the plasma of rats receiving a diet with the addition of Cr-Pic or Cr-Met was higher than in rats receiving a diet without added Cr or with the addition of Cr-NP (*P* < 0.001). In the plasma of rats receiving Cr-Pic, the insulin level was higher (*P* = 0.034) and the noradrenalin level was lower (*P* = 0.009) than in the rats from the other groups. The plasma of rats receiving added Cr, irrespective of its form, had a higher level of serotonin (*P* = 0.037) and a lower level of histamine (*P* < 0.001) (Table 3).

The rats receiving a diet with added Cr-Pic or Cr-NP had a lower plasma Ca level than the rats receiving a diet with Cr-Met (*P* = 0.003) and a lower P level (*P* < 0.001) than the rats in the other groups. A higher Mg level (*P* = 0.019) was noted in the plasma of rats receiving Cr-Pic than in rats from the groups receiving a diet without added Cr. In the rats receiving Cr-NP, the Fe level was higher than in rats receiving Cr-Pic (*P* = 0.044). A higher Zn level (*P* < 0.001) was noted in the plasma of rats receiving Cr-NP than in those receiving Cr-Pic or Cr-Met (Table 4).

## Discussion

The use of high-fat diet, especially for a long time, leads to impairment of carbohydrate and lipid metabolism and to obesity. Rats fed a HFD had higher body weight than rats fed a standard diet despite lower feed intake [26]. Obesity in rats fed a HFD may be due to the higher caloric value of the diet as well as to increased concentrations of leptin and reduced concentrations of ghrelin, as noted in our study. The decrease of ghrelin level in the plasma observed in obesity is most likely a physiological adaptation to the positive energy balance associated with obesity. Tschöp et al. [32], in a human study, found that the ghrelin level in the plasma is negatively associated with the degree of obesity. Beck et al*.* [33] and Beck and Richy [34], in a study in rats, also showed that an increase in the fat content of the diet results in reduced secretion of this hormone. Our study showed that the use of a HFD also increases leptin levels in the plasma. Ghrelin, known as the ‘hunger hormone’, and leptin, the ‘satiety hormone’, are negatively correlated. Low ghrelin levels are accompanied by high leptin levels, which is consistent with the results of the present study. Handjieva-Darlenska and Boyadjieva [35] also noted higher leptin levels and lower ghrelin levels in rats fed a high-fat diet than in the control group. Ghrelin can interact with leptin in the CNS, mainly at the level of the arcuate nucleus of the hypothalamus, in which both leptin and ghrelin receptors are present [33, 34]. Leptin is crucial in maintaining glucose homeostasis and is considered to be a factor inhibiting insulin secretion by pancreatic β cells [4, 36]. In our study, the use of a high-fat diet reduced the insulin level in the plasma of rats. This may be due to damage of pancreas. Our team's previous research [26] showed presence of extensive foci of steatosis in the pancreas of rats fed a high-fat diet. Tuzcu et al. [37] noted an increased insulin level (40 vs 33 pmol/l) in rats fed a HFD compared to rats receiving a standard diet. Sahin et al. [38] also report that a HFD increased the plasma insulin level.

Insulin secretion is additionally influenced by another hormone—serotonin. Paulmann et al. [39] noted that serotonin regulates insulin secretion via serotonylation of GTPases in pancreatic β cells. In our study, rats fed a HFD had higher serotonin and lower noradrenaline levels. Studies by Kim et al. [40] and Bertrand et al. [41] also showed higher serotonin levels in mice fed a HFD. Elevated levels of 5-HIAA, the main metabolite of serotonin, have also been observed in the plasma [42] and urine [43] of humans with obesity. Those two studies also showed a positive correlation between the fasting glucose concentration in the blood and 5-HIAA level. Rats fed a high-fat and high-cholesterol diet have higher expression of Tph1, and thus increased secretion of serotonin from the small intestine [44]. In turn, noradrenaline inhibits gene expression and reduces the level of circulating leptin in the body [4]. Carbohydrate metabolism is affected by histamine as well [45, 46]. Our study showed a reduced histamine level in rats receiving a high-fat diet compared to rats receiving a standard diet. Ji et al. [47] found that administration of the fat emulsion Liposyn II (20%) into the duodenum of rats increased the release of histamine into the intestinal lymph.

The use of a HFD in rats affected not only hormonal metabolism, but also the level of mineral elements in the blood plasma. The high-fat diet in the present study reduced the plasma levels of Cr, Ca, P, Mg and Zn. The lower level of these elements in the plasma may have been due to lower feed intake by rats fed a HFD as well as to increased excretion in the faeces. In addition, Ca, Mg and Zn ions combine with fatty acids to form water-insoluble calcium, magnesium or zinc soaps and are excreted in this form in the faeces [48].

One way to neutralise the negative effect of a HFD is to add Cr to the diet [24]. In the present study, a diet with added Cr, irrespective of its form, increased Cr intake from the diet as well as excretion of this element in the urine and faeces. Kottwitz et al. [49] noted that most of the Cr absorbed from CrCl_3_ is excreted in the urine within the first 2 days after it is ingested. Actual intestinal absorption (retention in the entire body and excretion in the faeces) of Cr from Cr-Pic is twice as high as from CrCl_3_, but a large proportion of absorbed Cr-Pic is found in the transport pool directed to excretion by the kidneys. Only a small portion of absorbed Cr-Pic is metabolised in the liver to the physiological form of Cr and stored in the body with a half-life of more than 100 days. For this reason, in the first 24 h after oral administration, most tissues (muscle, fat, bone and brain) show higher concentrations of ^51^Cr from CrCl_3_ than from Cr-Pic. In the present study, the use of a diet with the addition of organic forms of Cr resulted in greater Cr retention than in rats fed a diet with Cr-NP. According to Lien et al. [24], both nanoparticles of Cr-Pic (55–100 nm) and Cr-Pic administered to rat at dose of 300 μg kg^−1^ show high % digestibility. The higher % retention of Cr from Cr-Pic or Cr-Met in our study suggests that Cr in these forms is more easily digestible than in the form of Cr-NP, and a larger pool of it is retained in the body.

The use of a diet with added organic forms of chromium—Cr-Pic or Cr-Met—resulted in an increase in the plasma level of leptin in rats. Orhan et al. [1], who administered Cr-Pic and biotin or Cr-Hist and biotin to rats fed a HFD noted a reduction of the level of this hormone relative to rats fed a HFD without added Cr. Similarly, Inanc et al. [20] reported a decrease in leptin level in obese women who received chromium as Cr-Pic at 200 µg/day for 8 weeks. They did not, however, demonstrate an influence of Cr on the ghrelin or insulin level. Our study showed higher insulin level in the plasma of rats receiving Cr-Pic in their diet. Similarly, rats receiving a diet with Cr-acetate or Cr-glycinate had increased level of insulin in the blood [38]. According to Tuzcu et al. [37], rats fed a HFD supplemented with Cr-His had higher serum insulin level than rats fed a HFD without added Cr, while administration of Cr-His in the control group did not affect the insulin level. The role of Cr in insulin secretion is multi-faceted [50]. Most importantly, Cr increases the activity of 5′AMP-activated protein kinase, which plays a key role in the response to insulin, and insulin receptor kinase, thus enhancing insulin signalling [51, 52]. Chromium also induces translocation of glucose transporter 4 (GluT4) to the cell membrane, thereby promoting glucose metabolism [53].

The present study also showed that the addition of Cr to the diet of rats, irrespective of its form, increased the level of serotonin and reduced the level of histamine in the plasma of the rats. Franklin and Odontiadis [22], in a study on rats fed a diet supplemented with 100 mg/kg Cr in the form of Cr-Pic, also reported an increase in the serotonin level in the brain and increased sensitivity of central serotonin 2A receptors (5-HT2A). This is most likely linked to increased transport of the serotonin precursor tryptophan to the brain through the blood–brain barrier. This process is influenced by the level of tryptophan in the blood, its ratio to branched-chain amino acids (BCAA), and the insulin level [54, 55]. By promoting insulin secretion, chromium may also cause a decrease in the level of histidine, a histamine precursor. Moreover, histamine influences carbohydrate metabolism in the body by regulating the glucose level in the blood [45, 46]. In our study, only rats receiving Cr-Pic in their diet had a reduced level of noradrenaline. Franklin and Odontiadis [22] reported a higher level of noradrenaline in the brain of rats receiving 100 mg/kg Cr-Pic. Noradrenaline is one of the hormones that regulates lipolysis. A reduced noradrenaline level and increased insulin level in the plasma of rats receiving Cr-Pic may indicate inhibition of lipolysis. The addition of Cr to the diet of rats in the form of Cr-Pic or Cr-NP resulted in a decrease in the level of P and an increase in the concentration of Mg in the plasma of rats. This mechanism is not fully explained and requires further research.

## Conclusions

A high-fat diet was shown to negatively affect the level of hormones regulating carbohydrate metabolism (increasing leptin level and decreasing levels of ghrelin and insulin). Cr in organic forms was found to be better retained in the body of rats than Cr in nanoparticles form. However, Cr-Pic was the only form that increased the insulin level, which indicates its beneficial effect on carbohydrate metabolism. Rats fed a high-fat diet had an increased level of serotonin and a reduced level of noradrenaline. The addition of Cr to the diet, irrespective of its form, also increased the serotonin level, which should be considered a beneficial effect. Rats fed a high-fat diet had an unfavourable reduction in the plasma concentrations of Ca, P, Mg and Zn. The reduction in P in the plasma induced by supplementation with Cr in the form of Cr-Pic or Cr-NP may exacerbate the adverse effect of a high-fat diet on the level of this element.

## Data Availability

All relevant data are within the paper and its supporting information files. Additional data are available on request from the corresponding authors.
